# Further evidence that far‐UVC for disinfection is unlikely to cause erythema or pre‐mutagenic DNA lesions in skin

**DOI:** 10.1111/phpp.12580

**Published:** 2020-06-12

**Authors:** Isla Rose Mary Barnard, Ewan Eadie, Kenneth Wood

**Affiliations:** ^1^ SUPA School of Physics & Astronomy University of St Andrews St Andrews UK; ^2^ Photobiology Unit NHS Tayside Medical Physics Ninewells Hospital and Medical School Dundee UK

To The Editor

It is well understood that ultraviolet‐C (UVC) radiation is effective for the destruction of micro‐organisms and drug‐resistant bacteria and is being investigated for its effectiveness at destroying the virus responsible for the current COVID‐19 global pandemic.^1^, ^2^, ^3^, ^4^

Far‐UVC (200‐220 nm) has been proposed as an effective disinfection radiation that is safe to humans.^5^ In 2014, Woods et al undertook a first‐in‐person study to assess the effect on skin of a 222 nm UVC emitting device (Sterilray disinfectant wand, Healthy Environment Innovations, Dover, NH, USA).^6^ The study concluded that erythema was induced at radiant exposures lower than required for the threshold bacteriostatic effect. Direct CPD formation was also observed in both the supra‐basal layer (all four volunteers) and basal layer (2 out of 4 volunteers) of the volunteer skin. Woods et al hypothesised that a small amount of longer wavelength UVC radiation above 250 nm (<3%) may be contributing to the observed effects.^6^ We wished to determine why these results contrast with other published studies investigating far‐UVC sources.^2^, ^5^

To determine the depth penetration of UVC in Fitzpatrick Skin Type I and the associated direct CPD formation, we employ Monte Carlo radiation transfer (MCRT) codes previously used to study ultraviolet radiation transport in skin.^7^, ^8^ The MCRT simulation initiates UV power packets from the spectrum of the source, that diffusely irradiate the skin, and follows their subsequent random walk through a three‐dimensional grid comprising 10^6^ cubic voxels representing a 0.4 mm thick 5‐layer skin model.^7^ The wavelength‐dependent absorption and scattering properties for the different layers are adapted from van Gemert et al^9^ The simulation outputs fluence as a function of wavelength within different layers of the skin. To determine the probability of producing CPD, relative to 260 nm in the upper epidermis, we combine the computed fluence rates with the CPD yield spectrum from Matsunaga et al, covering the wavelength range 200 nm to 365 nm, and the concentration of DNA in each skin layer.^10^

**FIGURE 1:**
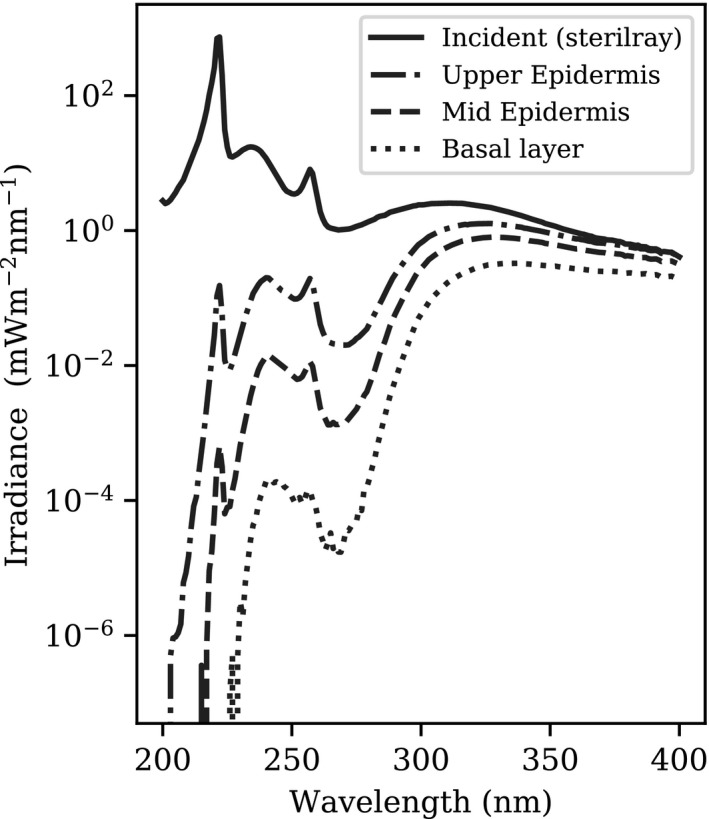
Irradiance of the far‐UVC source investigated by Woods et al^6^ and MCRT simulated fluence incident on the upper and mid‐epidermis and basal layer

Figure 1 shows the spectral irradiance of the 222 nm UVC source incident on the skin and the resulting spectral fluence incident on the upper and mid‐epidermis and on the basal layer. In this simulation, no radiation from the 222 nm peak reaches the basal layer. Lower intensity incident irradiances above 270 nm, barely seen in the original non‐logarithmic spectrum, penetrate to the basal layer.

**FIGURE 2:**
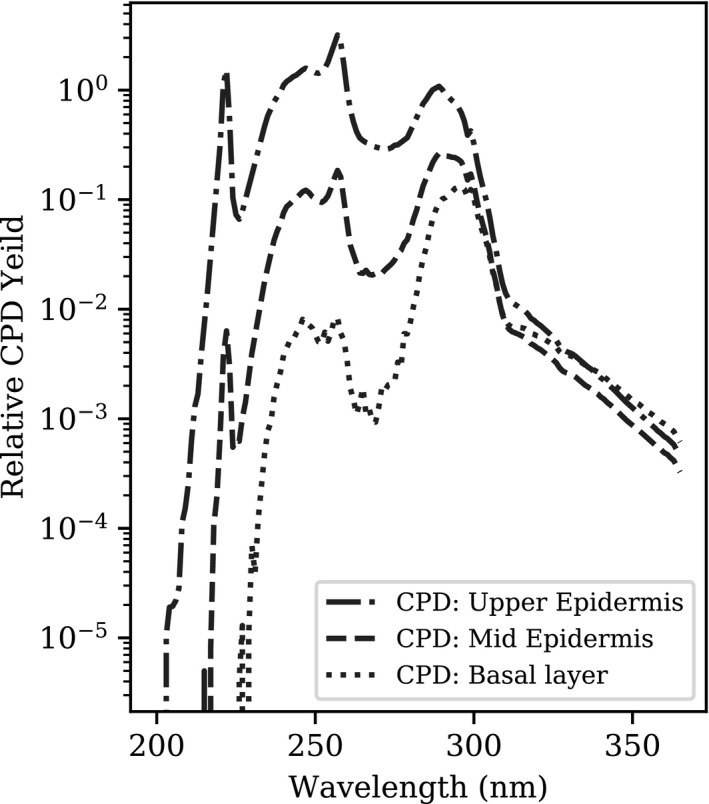
Spectral probability of producing CPD, relative to 260 nm in the upper epidermis, from the far‐UVC source. Three layers of the skin are shown: the upper epidermis, mid‐epidermis and basal layer

Figure 2 shows the probability of producing CPD, relative to 260 nm, in the upper and mid‐layers of the epidermis and in the basal layer. CPD formation in the basal layer from this 222 nm UVC source is most probable from low intensity wavelengths of irradiation between 270 nm and 310 nm. CPD from shorter wavelengths is seen in the upper and mid‐epidermis.

Our results demonstrate that whilst a percentage of far‐UVC radiation at 222 nm penetrates to the upper epidermis, minimal reaches the mid‐epidermis and none in the basal layer. Direct CPD formation in the basal layer observed by Woods et al is likely to have arisen from very low intensity source emissions above 230 nm, in particular the 270 nm to 310 nm wavelength range, where the spectral emissions are not visualised without plotting incident irradiance on a logarithmic scale. Careful filtering of UVC spectral emissions, to remove unwanted longer wavelengths, has been shown not to induce tissue inflammation or increase pre‐mutagenic DNA lesions in both mammalian skin and an in vitro human skin model.^2^, ^5^, ^11^ Whilst initially this would appear to contradict our results, CPD formation in the upper and mid‐layers of the epidermis, from wavelengths below 230 nm, is of minimal contribution to overall simulated CPD (6.4% and 0.3% for each layer, respectively). This may explain why, at clinically relevant radiant exposures, filtered far‐UVC has not resulted in CPD formation but does not rule out tissue inflammation as a result of severe overexposure.

The results of our simulations, combined with additional published evidence, indicate that further investigation of far‐UVC for human skin disinfection should not be dissuaded by the results from Woods et al, with in vivo human studies being required to definitively answer the question.
